# Entropy Production Associated with Aggregation into Granules in a Subdiffusive Environment

**DOI:** 10.3390/e20090651

**Published:** 2018-08-30

**Authors:** Piotr Weber, Piotr Bełdowski, Martin Bier, Adam Gadomski

**Affiliations:** 1 Atomic and Optical Physics Division, Department of Atomic, Molecular and Optical Physics, Faculty of Applied Physics and Mathematics, Gdańsk University of Technology, Narutowicza 11/12, 80-233 Gdańsk, Poland; pioweber@pg.edu.pl or; 2 Institute of Mathematics and Physics, UTP University of Science and Technology, Kaliskiego 7, 85-796 Bydgoszcz, Poland; adam.gadomski@utp.edu.pl or; 3 Deptartment of Physics, East Carolina University, Greenville, NC 27858, USA

**Keywords:** entropy production, fractional calculus, aggregation

## Abstract

We study the entropy production that is associated with the growing or shrinking of a small granule in, for instance, a colloidal suspension or in an aggregating polymer chain. A granule will fluctuate in size when the energy of binding is comparable to $k_{B}T$, which is the “quantum” of Brownian energy. Especially for polymers, the conformational energy landscape is often rough and has been commonly modeled as being self-similar in its structure. The subdiffusion that emerges in such a high-dimensional, fractal environment leads to a Fokker–Planck Equation with a fractional time derivative. We set up such a so-called fractional Fokker–Planck Equation for the aggregation into granules. From that Fokker–Planck Equation, we derive an expression for the entropy production of a growing granule.

## 1. Introduction

Granules commonly occur in soft materials such as gels and biopolymer complexes. Granules are of the essence in colloidal suspensions. The size of an individual granule can fluctuate when binding energies are of a $k_{B}T$ order of magnitude and too small for the formation of rigid crystals [1]. The folded part of a protein can also be thought of as a granule. Even in their native state, many proteins are in large part still unfolded random coils. Such proteins sensitively rely on the right folding for their optimal functioning. Below, we study the random Brownian fluctuations of granule sizes.

The aggregation of segments of hyaluronic acid into granules is an example with biomedical significance. Hyaluronic acid is a biopolymer that is present in large concentration in the synovial fluid [2,3]. In a healthy synovial joint, the synovial fluid reduces friction and absorbs shocks. Knees, wrists, hips, and shoulders all fall into the category of synovial joints [4]. For the synovial fluid to exhibit optimal viscoelasticity, the hyaluronic acid needs to have the right amount of aggregation. Understanding the physics of the growing and shrinking of granules can be important for the understanding and treatment of many types of arthritis [3].

In this article, we take a very abstract approach that follows the work of Frauenfelder [5]. We view the polymer as a chain with *N* degrees of freedom, where *N* is a large number. Frauenfelder and his co-workers treated the (N−1)-dimensional energy hypersurface as a fractal-like structure where barrier heights follow a power-law distribution. With such a structure, the times to transit from one minimum to another are no longer exponentially distributed, but, instead, also follow a power law. In the case of a high-dimensional, self-similar energy landscape, the protein furthermore no longer diffuses “normally” in its conformational space (i.e., following $\langle x^{2}\left(t\right)\rangle \propto t$, where *x* is the diffused distance and *t* is the time), but instead performs so-called subdiffusion [6,7], i.e.,

(1) $$\langle x^{2}\left(t\right)\rangle \propto t^{\alpha },\;\mathrm{where}\;0<\alpha <1.$$

The idea of a fractal energy landscape has been very fruitful in explaining kinetic properties of proteins [8,9].

With a fractal topology of the energy landscape, we can discriminate between slower and faster timescales. There are parts of the molecule that do not change their configuration during an experiment or in the course of many steps in a simulation. The set of slower degrees of freedom can be considered as giving rise to stable structures, i.e., “granules”. The picture that ultimately arises is one of a flexible chain in which the granules are similar to “beads”. As time evolves, the volumes of these beads can increase or decrease. Because the beads are joined, such aggregation dynamics are different from ordinary crystallization [1,10].

Below, we introduce a density f(v,t), such that f(v,t)dv is the probability that a grain has a volume between *v* and v+dv. When the system is out-of-equilibrium, f(v,t) will change in time and move towards an equilibrium distribution $f_{eq}\left(v\right)$. In a mesoscopic environment, f(v,t) will change due to deterministic forces and due to Brownian fluctuations, i.e., diffusion. Such setups lead to a partial differential equation for the time evolution of f(v,t). This is the well-known Fokker–Planck Equation [11]. For normal diffusion, this equation is first order in *t* and second order in *v*. We show below how in a subdiffusive environment, the Fokker–Planck Equation involves fractional derivatives (i.e., operators of the form $d^{\alpha }/dx^{\alpha }$, where α is noninteger). Such fractional derivatives become meaningful and analytically manageable only after a Laplace or Fourier transformation turns a derivative into a multiplicative factor. Nevertheless, fractional derivatives and integrals have found many applications in various fields of science and engineering [12]. Ultimately, we use the fractional Fokker–Planck Equation to derive a formula for the entropy production in a subdiffusive environment. Such entropy production is a measure for the speed of the process.

## 2. Results

In our analysis, we take the volume of the granule as the degree of freedom. Because that volume is subject to Brownian fluctuations, it is a stochastic variable. When the system is not at equilibrium, the distribution f(v,t) will change in time and move towards the equilibrium distribution. As grains do not appear or disappear, but only grow or shrink, we have a Continuity Equation,

(2) $$\frac{\partial f(v,t)}{\partial t}=-\frac{\partial J(v,t)}{\partial v},$$

where J(v,t) is the rate at which particles of volume *v* are changing their volume. In a mesoscopic environment, f(v,t) changes due to deterministic forces and due to Brownian fluctuations, i.e., diffusion. For J(v,t) we thus have the formula [1]:

(3) $$J\left(v,t\right)=-\tau v^{\gamma -1}f\left(v,t\right)-Dv^{\gamma }\frac{\partial }{\partial v}f\left(v,t\right),$$

where *D* is the diffusion coefficient and τ represents the surface tension between the granule and the surrounding solution. To come to the first term on the right hand side, we realize that the surface energy between a granule and the surrounding aqueous solution is proportional to the surface area of the granule and therefore proportional to $v^{\gamma }$, where γ is close to 2/3. Deviations from the 2/3 value occur when the growth or shrinkage is anisotropic. Taking the derivative of the surface energy with respect to *v*, we come to the factor $-\tau v^{\gamma -1}$ which represents the force towards volume increase. The second term on the right hand side of Equation (3) is the diffusive term, where *D* is the diffusion coefficient. A volume change of a granule occurs when attachment or detachment occurs at the surface of that granule. It is therefore that the diffusive term is proportional to the surface of the grain. Numerical constants due to, among other things, the particular shape of the granule, can be absorbed in the constant parameters τ, γ, and *D*.

The traditional Fokker–Planck Equation for the case of “normal” diffusion is obtained by substituting Equation (3) into Equation (2). The result is a partial differential equation that is first order in the time *t* and second order in the volume *v*.

The most concise and elegant way to account for the effect of the anomalous subdiffusion is to follow Equation (1) and perform a nonlinear power-law transformation for the time: $t\to t^{\alpha }$. This leads to a new Continuity Equation [13,14,15],

(4) $$\frac{\partial f(v,t)}{\partial t}=-{\;}_{0}D_{t}^{\beta }\frac{\partial J(v,t)}{\partial v},$$

where

(5) $${}_{0}D_{t}^{\beta }f\left(t\right)=\frac{1}{\Gamma (1-\beta )}\left(\frac{\partial }{\partial t}\right)\int_{0}^{t}\frac{f(u)}{{\left(t-u\right)}^{\beta }}du$$

is the so-called Riemann–Liouville fractional derivative [16]. We, furthermore, have β=1−α. Thus, 0<α<1 translates into 0<β<1. Realizing that derivatives can be taken in any order and by taking a modified current,

(6) $$\tilde{J}\left(v,t\right)={\;}_{0}D_{t}^{\beta }J\left(v,t\right),$$

we can see that the subdiffusive form of the Continuity Equation effectively only involves a modification of the current:

(7) $$\frac{\partial f(v,t)}{\partial t}=-\frac{\partial }{\partial v}\tilde{J}\left(v,t\right).$$

An associated Fokker–Planck Equation can be obtained by taking the appropriate fractional derivatives with respect to time on the right-hand-side of Equation (3).

Next, we go back to the case of normal diffusion. For the case described by Equations (2) and (3), the Shannon entropy of the system is given by [10,17]:

(8) $$S=S_{eq}-k_{B}\int f\left(v,t\right)\ln\frac{f(v,t)}{f_{eq}\left(v\right)}dv.$$

Here, $f_{eq}\left(v\right)$ is the equilibrium distribution and $k_{B}$ is Boltzmann’s constant. At equilibrium, we have J(v,t)=0. The entropy of the equilibrium state is given by $S_{eq}$. The second term on the right-hand-side of Equation (8) can be thought of as a “conditional entropy” associated with a non-equilibrium density f(v,t) [18]. This conditional entropy represents a kind of “distance” of the non-equilibrium distribution f(v,t) from the equilibrium distribution $f_{eq}\left(v\right)$. The distribution f(v,t) is normalized. Thus, the variation of the entropy, δS, has a simple one-term dependence on a variation, δf(v,t), in the distribution f(v,t) [19]:

(9) $$\delta S=-k_{B}\int \delta f\left(v,t\right)\ln\frac{f(v,t)}{f_{eq}\left(v\right)}dv.$$

With the Continuity Equation and using partial integration (whereby it should be realized that f(v,t) vanishes at v→0 and v→∞), we next find

(10) $$\sigma \left(t\right)=\frac{dS}{dt}=-k_{B}\int J\left(v,t\right)\frac{\partial }{\partial v}\left[\ln\frac{f(v,t)}{f_{eq}\left(v\right)}\right]dv$$

for the entropy production that is associated with a flux J(v,t) [1]. Following Onsager, we can interpret the entropy production as a product of a flux J(v,t) and a thermodynamic force $k_{B}\partial \ln\left[f\left(v,t\right)/f_{eq}\left(v\right)\right]/\partial v$.

Using Equation (6), it is straightforward to formulate a subdiffusive equivalent of Equation (10):

(11) $$\tilde{\sigma }\left(t\right)=-k_{B}\int \tilde{J}\left(v,t\right)\frac{\partial }{\partial v}\left[\ln\frac{f(v,t)}{f_{eq}\left(v\right)}\right]dv.$$

This result is easy to intuit: thermodynamic forces are not affected when going from a “normal” environment to a subdiffusive environment. The fluxes, however, are affected. Finally, we mention how two well-known formulas that apply in the case of normal diffusion easily extend to the subdiffusive case. The product

(12) $${\tilde{J}}_{S}\left(v,t\right)=k_{B}\tilde{J}\left(v,t\right)\ln\frac{f(v,t)}{f_{eq}\left(v\right)}$$

can be interpreted as the entropy flow [1] and, when close to equilibrium, a linear force–flux relation applies, i.e.,

(13) $$\tilde{J}\left(v,t\right)=-L\left(v\right)k_{B}\frac{\partial }{\partial v}\ln\frac{f(v,t)}{f_{eq}\left(v\right)},$$

where L(v) is a scalar (Onsager type) coefficient.

## 3. Discussion and Conclusions

In a subdiffusive setting, the Fokker–Planck Equation contains fractional derivatives. We have shown in detail how to construct such a fractional Fokker–Planck Equation for the case of the growing and shrinking of mesoscopic granules. Though fractional derivatives are hard to intuit and present serious mathematical complications, the associated thermodynamics and statistical physics remain straightforward. By going from the “normal” to the subdiffusive case through a straightforward, nonlinear, power-law transformation of the time variable, we avoided mathematical complications. We have seen how, ultimately, an ordinary force–flux relation still applies. Only the flux terms undergo slight modification.

Over the past decade, many cases of subdiffusion have been identified. It turns out, for instance, that anomalous diffusion is almost the rule inside a living cell [20]. Anomalous diffusion and fractional derivatives have also been used to describe fluid flow in porous media such as soils [21,22].

The proper “granulization” of a polymer chain is of critical importance in many biochemical processes. The aggregation into granules generally exhibits a very sensitive dependence on parameters like electrolyte concentration, temperature, etc. We already mentioned how our formalism applies in the case of hyaluronic acid. The correct folding and unfolding of DNA and proteins is essential for many of life’s processes. Many ailments, such as prion diseases, are associated with the misfolding of proteins. The formalism we have presented may guide the way to a more fundamental and more quantitative understanding of these biological processes.
